# Dysregulated nitric oxide signaling as a candidate mechanism of fragile X syndrome and other neuropsychiatric disorders

**DOI:** 10.3389/fgene.2014.00239

**Published:** 2014-07-22

**Authors:** Steven M. Colvin, Kenneth Y. Kwan

**Affiliations:** Department of Human Genetics – The Molecular and Behavioral Neuroscience Institute, University of Michigan Medical SchoolAnn Arbor, MI, USA

**Keywords:** cerebral neocortex, pyramidal neurons, neurodevelopmental disorders, nitric oxide signaling, Broca’s speech and language area, anterior cingulate cortex, neural circuit assembly, human fetal brain

## Abstract

A mechanistic understanding of the pathophysiology underpinning psychiatric disorders is essential for the development of targeted molecular therapies. For fragile X syndrome (FXS), recent mechanistic studies have been focused on the metabotropic glutamate receptor (mGluR) signaling pathway. This line of research has led to the discovery of promising candidate drugs currently undergoing various phases of clinical trial, and represents a model of how biological insights can inform therapeutic strategies in neurodevelopmental disorders. Although mGluR signaling is a key mechanism at which targeted treatments can be directed, it is likely to be one of many mechanisms contributing to FXS. A more complete understanding of the molecular and neural underpinnings of the disorder is expected to inform additional therapeutic strategies. Alterations in the assembly of neural circuits in the neocortex have been recently implicated in genetic studies of autism and schizophrenia, and may also contribute to FXS. In this review, we explore dysregulated nitric oxide signaling in the developing neocortex as a novel candidate mechanism of FXS. This possibility stems from our previous work demonstrating that neuronal *nitric oxide synthase 1* (*NOS1* or *nNOS*) is regulated by the FXS protein FMRP in the mid-fetal human neocortex. Remarkably, in the mid-late fetal and early postnatal neocortex of human FXS patients, NOS1 expression is severely diminished. Given the role of nitric oxide in diverse neural processes, including synaptic development and plasticity, the loss of NOS1 in FXS may contribute to the etiology of the disorder. Here, we outline the genetic and neurobiological data that implicate neocortical dysfunction in FXS, review the evidence supporting dysregulated nitric oxide signaling in the developing FXS neocortex and its contribution to the disorder, and discuss the implications for targeting nitric oxide signaling in the treatment of FXS and other psychiatric illnesses.

## INTRODUCTION

Although a single locus genetic disorder, fragile X syndrome (FXS), the leading inherited cause of intellectual disability (ID) and monogenic syndromic form of autism spectrum disorder (ASD), remains incompletely understood at the mechanistic level. Since the identification of *FMR1* as the X-linked gene affected in FXS (Warren et al., 1987; Oberlé et al., 1991; Verkerk et al., 1991; Vincent et al., 1991), significant research efforts have focused on FMRP, the protein encoded by *FMR1* (Ashley et al., 1993). FMRP is an RNA-binding protein that controls the localization, stability, and translation of neuronal mRNAs (Bagni and Greenough, 2005; Zalfa et al., 2007; Bassell and Warren, 2008). It is highly expressed in neurons and nearly ubiquitously present in the central nervous system (Devys et al., 1993; Hinds et al., 1993; Bakker et al., 2000; O’Donnell and Warren, 2002). Because FMRP is expressed in the brain without preference for particular regions or neuronal subtypes, its expression pattern does not provide insights into the potential neural or neuronal substrates underlying the intellectual and behavioral deficits of the disorder. At the molecular level, FMRP interacts with and controls the translation of a large number of neuronal mRNAs (Brown et al., 2001; Darnell et al., 2011), estimated to be more than 800 transcripts. Consistent with the significant localization of FMRP to dendritic spines (Feng et al., 1997; Antar et al., 2004; Gabel et al., 2004), which are specializations at post-synaptic sites, many of these transcripts play key roles in synapse formation and function (Brown et al., 2001; Darnell et al., 2001, 2011; Miyashiro et al., 2003; Kindler and Kreienkamp, 2012). The converging localization and function of many FMRP target mRNAs to the synapses has illuminated FMRP neurobiology; however, precisely which of the myriad transcripts are mechanistically most significant for the psychopathology of FXS, or most suitable as targets of molecular therapeutics, remains to be fully explored.

One potential mechanism, coined the metabotropic glutamate receptor (mGluR) theory of FXS, was proposed following observations linking mGluR signaling, long-term depression (LTD), protein synthesis, and FMRP function (Weiler et al., 1997; Huber et al., 2000, 2001, 2002) and has been a focus of recent basic and clinical research (Bear et al., 2004; Dölen et al., 2007; Ronesi and Huber, 2008; Berry-Kravis, 2014). At the time of writing, drugs targeting several molecular players in the mGluR signaling pathway are being studied in clinical trials (Berry-Kravis, 2014; Erickson et al., 2014; Jacquemont et al., 2014; Pop et al., 2014).

Since its original proposal, several lines of converging evidence have corroborated the mGluR theory. The majority of the data supporting excessive LTD in FXS is derived from studies of the hippocampus (Huber et al., 2002; Bear et al., 2004), a critical brain region for learning and memory. The specific contribution of overactive hippocampal LTD to the pathophysiology of FXS, however, remains incompletely explored. Furthermore, the precise identities of the *de novo* proteins necessary for LTD have remained largely mysterious, and they are likely to represent only a subset of FMRP targets. Therefore, it is possible that additional mechanisms or brain regions are involved in the intellectual and behavioral manifestations of FXS. Elucidating these mechanisms is expected to uncover the neurobiology pertinent to FXS and provide novel targets toward molecular therapy.

In this review, we explore disrupted nitric oxide (NO) signaling as a new candidate mechanism of FXS centered in the developing neocortex. In our recent work, we showed neuronal *nitric oxide synthase 1* (*NOS1* or *nNOS*) to be translationally regulated by FMRP in the fetal human neocortex and severely reduced in the fetal and post-natal developing neocortex of FXS patients (Kwan et al., 2012a). As NO is a multifunctional signaling molecule with diverse neural functions, including synapse development, neural transmission, and synaptic plasticity, the disruption of NOS1 protein translation in FXS may underlie the pathogenesis of the disorder. Here, we (1) outline the genetic and neurobiological data that implicate neocortical dysfunction in the etiology of FXS; (2) review the evidence supporting dysregulated NO signaling in the developing FXS neocortex and its potential functional contribution to the disorder; and (3) discuss the implications for targeting NO signaling in the treatment of FXS and other psychiatric disorders, including autism and schizophrenia (SCZ).

## NEOCORTICAL DYSFUNCTION AS A NEURAL BASIS OF FXS

Research into the mechanisms underlying FXS has long been focused on the biology of FMRP, a key aspect of which is centered on the identities of its mRNA targets (Brown et al., 2001; Darnell et al., 2011). Although the number of direct FMRP-interacting mRNAs is numerous and their individual contributions to the disorder remain largely unknown, some neurobiological aspects behind their function and dysfunction have emerged. FMRP regulates the translation of many transcripts encoded by genes implicated in ASD (Darnell et al., 2011), and *de novo* mutations in ASD simplex families preferentially affect genes encoding FMRP targets (Iossifov et al., 2012). This convergence suggests that the pathophysiologies of ASD and FXS may be mediated by common mechanisms, and is consistent with the broad behavioral overlap between FXS and autism (Rogers et al., 2001; Hagerman et al., 2010). Similarly, genes disrupted by *de novo* variants in SCZ are also significantly enriched for those that encode FMRP target transcripts (Fromer et al., 2014; Purcell et al., 2014). Although a potential link between FXS and SCZ has not been extensively explored, decreased FMRP levels have been associated with intellectual deficits and earlier onset in SCZ (Kovács et al., 2013). Furthermore, recent findings have shown that the genetic underpinnings of ASD and SCZ exhibit significant overlap (Carroll and Owen, 2009; McCarthy et al., 2014). This putative mechanistic intersection between FXS, ASD, and SCZ implicates a partial convergence in the molecular and neural substrates underlying these disorders.

**FIGURE 1:**
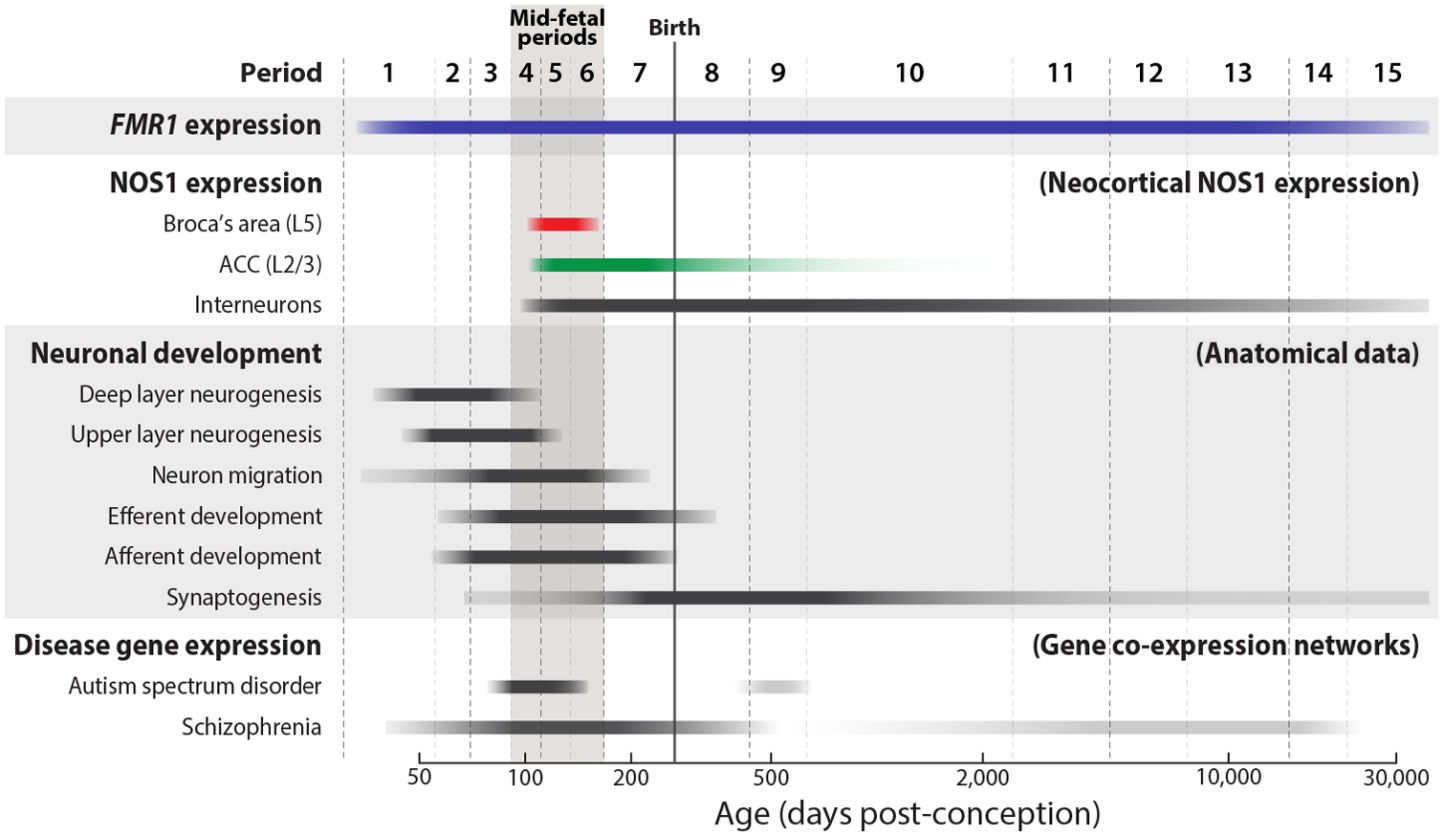
** Trajectories of NOS1 and *FMR1* expression and key developmental events in the mid-fetal human neocortex.** The approximate timing and duration of selected neocortical developmental processes, as defined by anatomical findings, are illustrated (Bourgeois, 1997; de Graaf-Peters and Hadders-Algra, 2006; Clancy et al., 2007; Workman et al., 2013). *FMR1* (blue) is widely expressed in the brain from early fetal development into adult aging. During the mid-fetal ages, periods 4–6 (Kang et al., 2011), NOS1 is expressed in the pyramidal neurons of Broca’s area L5 (red) and ACC L2/L3 (green) (Kwan et al., 2012a). Unlike its transient pyramidal expression, NOS1 is expressed in interneurons from fetal development to late adulthood. Supporting a potential role in neuropsychiatric disorders, mid-fetal neocortical development has been recently implicated in analyses of gene co-expression networks of ASD- and SCZ-associated genes (Gulsuner et al., 2013; Parikshak et al., 2013; Willsey et al., 2013).

Because FMRP is expressed in most neuronal subtypes from early fetal development to aging (Abitbol et al., 1993; Devys et al., 1993; Hinds et al., 1993; Kang et al., 2011), its spatiotemporal expression pattern does not implicate any particular neural structure or developmental stage to be especially important to the etiology of the disorder. Recent advances in the fields of ASD and SCZ genetics have, however, begun to reveal the neurobiology and neural substrates that underlie complex disorders of brain development. Historically, the genetic heterogeneity of ASD, SCZ, and other psychiatric diseases has been a major challenge in identifying the genetic loci that carry definitive genetic risk. Ironically, the large number of risk loci that has impeded progress may now provide an opportunity to better pinpoint the neural substrates and developmental stages that are central to the pathophysiology of these disorders (State and Šestan, 2012). Because of the pleiotropic nature of gene function, the majority of genes in mammalian genomes are expressed in multiple organs over multiple time points during the development, maturation, and aging of the organism. Accordingly, expression analyses of ASD or SCZ risk genes using comprehensive spatiotemporal transcriptomic data from the human brain (Johnson et al., 2009; Colantuoni et al., 2011; Kang et al., 2011), have revealed highly disparate spatial patterns and developmental trajectories. However, gene co-expression network analyses, which can be used to identify clusters of highly correlated genes (Eisen et al., 1998; Stuart et al., 2003; Geschwind and Konopka, 2009), have uncovered key points of convergence in the timing and location of risk gene expression. Expression network analyses of high-confidence ASD loci have identified the mid-fetal neocortex to be a significant point of spatiotemporal intersection (Parikshak et al., 2013; Willsey et al., 2013), suggesting a significant contribution of the developing neocortex to ASD circuit dysfunction (Kwan, 2013). In SCZ, a similar analysis has also implicated the fetal frontal neocortex (Gulsuner et al., 2013). Mid-gestation is a critical developmental period during which key neocortical axon tracts are being established prior to the onset of significant synaptogenesis (**Figure 1**; Kang et al., 2011). This convergence of ASD and SCZ gene expression, therefore, strongly implicates miswiring of neocortical circuits in the pathophysiology of these neurodevelopmental disorders.

Neocortical dysfunction may similarly contribute to FXS etiology. The neocortex underlies conscious experience, thoughts, and actions in mammals, and is the seat of higher cognitive functions including speech and language and social cognition (Geschwind and Rakic, 2013), which are often affected in FXS and other neurodevelopmental disorders. The proper functioning of the neocortex depends on the precise wiring of its neural circuits during fetal and early post-natal development. Dysregulation of neocortical circuit assembly and function has long been thought to contribute to neuropsychiatric disorders. In FXS, cognitive and language development are delayed or impaired (Abbeduto et al., 2007), phenotypes that are consistent with disrupted neocortical function. With 30% of FXS patients meeting the full diagnostic criteria for autism and an additional 30% falling elsewhere on the autism spectrum (Harris et al., 2008), deficits in social cognition, another higher function centered in the neocortex, are also a key impairment in FXS. Anatomically, this possibility is further supported by findings that revealed altered dendritic spine morphologies in the neocortical pyramidal neurons of human FXS patients and *Fmr1* null mice (Comery et al., 1997; Irwin et al., 2000).

With recent advances in neuroimaging, deficits in neural connectivity are being mapped and alterations in neocortical circuitry have been reported in multiple disorders, including ASD, SCZ, and bipolar disorder (Kuperberg et al., 2008; Henin et al., 2009; Henseler et al., 2010; Hall et al., 2013; Zikopoulos and Barbas, 2013). Functional and anatomical imaging studies have also supported neocortical dysfunction in FXS. One analysis found that, during the counting Stroop interference task, normal activation in the inferior/middle frontal gyrus and inferior/superior parietal lobe was absent, and an aberrant activation in the anterior region of the prefrontal cortex (PFC) was present instead (Tamm et al., 2002). In the Go/NoGo task, FXS patients exhibited abnormal activation patterns in multiple brain regions, including significantly reduced activation in the supplementary motor area and anterior cingulate cortex (ACC; Menon et al., 2004). Furthermore, white-matter abnormalities were found in frontostriatal circuitry (Haas et al., 2009), and a recent analysis of functional connectivity and large-scale brain networks in FXS revealed widespread reductions in neocortical connectivity (Hall et al., 2013). Interestingly, maturation of the PFC was found to be aberrant in adolescent FXS patients, an alteration that is correlated with delayed cognitive maturation (Bray et al., 2011). The PFC, often considered to be the prototypical center of higher-order cognitive processing, is critical to working memory, executive function, and inhibition, and has been robustly implicated in multiple psychiatric disorders (Weinberger et al., 1986; Goldman-Rakic, 1996; Miller et al., 2002). Together, these lines of evidence converge on the possibility that alterations in neocortical circuitry underlie some aspects of FXS pathogenesis. The molecular mechanisms that may contribute to these alterations in FXS, however, have not been extensively explored.

## FMRP REGULATION OF NOS1 TRANSLATION IN THE HUMAN FETAL NEOCORTEX

Recently, we identified neuronal *NOS1* or *nNOS* to be a novel FMRP target mRNA in the mid-fetal human neocortex (Kwan et al., 2012a). This interaction was mediated by two G-quartet motifs within the coding region of human *NOS1* that are necessary and sufficient for FMRP binding. Consistent with a direct interaction, FMRP was found to be a positive regulator of NOS1 translation, controlling NOS1 protein levels in a dose-dependent manner *in vitro* and *in vivo*. FMRP is mostly known to be a translation repressor through a ribosome stalling mechanism that was elegantly shown using an *in vitro* system (Darnell et al., 2011). In some cases, it has been shown to function as a positive translational regulator (e.g., *SOD1*, *ASCL1* (*hASH1*), *Kcnd2* (*Kv4.2*), and *DLG4* (*PSD95*)) (Todd et al., 2003; Bechara et al., 2009; Fähling et al., 2009; Gross et al., 2011), and may mediate this translational control in a context- or activity-dependent manner.

Interestingly, FMRP binding and regulation of *NOS1* is species-dependent, being present in primate species, which exhibit conserved G-quartet sequences in *NOS1*, but absent in rodent species, which lack the *NOS1* G-quartets. Consistent with this species difference, *NOS1* has not been previously identified in the mouse brain to be an FMRP target mRNA (Brown et al., 2001; Darnell et al., 2011). The absence of the G-quartet motifs from rodent *Nos1* and the high degree of amino acid identity in FMRP among mammals suggest that the interaction between FMRP and *NOS1* evolved via changes in the *NOS1* sequence, as opposed to changes in FMRP function. This suggests the possibility of additional examples of evolutionary changes that can impact FMRP interaction with target mRNAs. Indeed, consistent with this possibility, a recent analysis of brain-expressed exons under purifying selection demonstrated enrichment for ASD-associated genes and ASD-relevant FMRP targets (Uddin et al., 2014).

**FIGURE 2:**
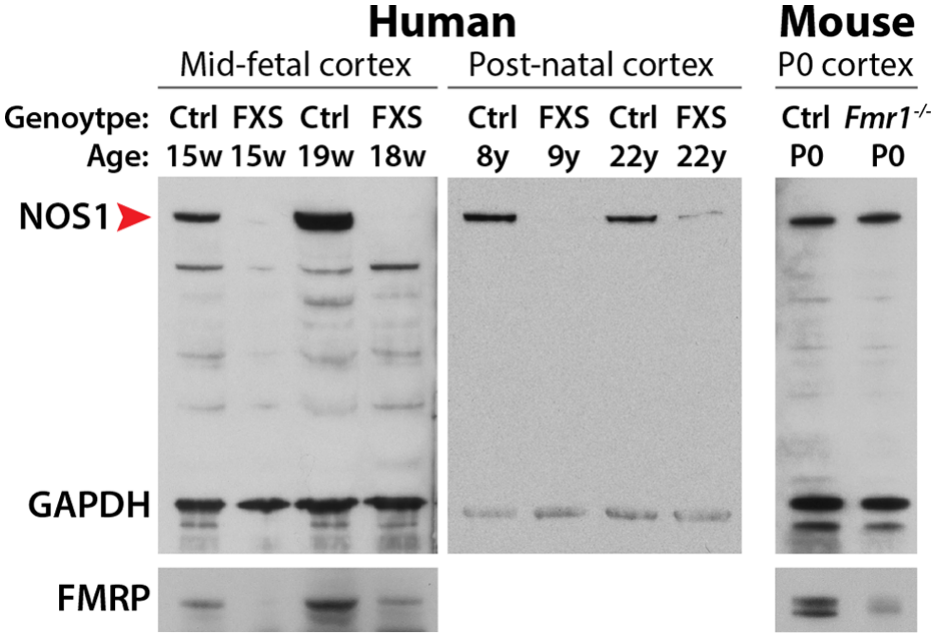
**Immunoblot analysis of NOS1 expression in the developing neocortex.** In the developing neocortex, the expression levels of NOS1 (red arrowhead) are severely reduced in human FXS patients. This loss is species-dependent. NOS1 levels are unaltered in the neocortex of neonatal *Fmr1* null mice, an animal model of FXS. Adapted from Kwan et al. (2012a).

The dose-dependence upregulation of NOS1 translation by FMRP in the normal human neocortex suggests the possibility of disrupted NOS1 protein synthesis in human FXS patients. Examination of FXS patient brains revealed, remarkably, that NOS1 is absent from the neocortex at fetal ages, and significantly decreased in the neocortex during postnatal development (**Figure 2**). Although a large number of FMRP target mRNAs have been identified in the mouse brain (Brown et al., 2001; Darnell et al., 2011), the majority of these transcripts have not been assessed for potential translational dysregulation in FXS patient materials. Therefore, *NOS1* represents the first mRNA that is found to interact with FMRP in the fetal human brain and translationally disrupted in the developing FXS patient brain. NOS1 is an enzyme that mediates the tightly-regulated biosynthesis of NO, a multifunctional gaseous signaling molecule (Bredt and Snyder, 1994; Garthwaite, 2008). Given the key role of NO in diverse neural processes including synaptic plasticity, retrograde signaling, development, excitotoxicity, and mental health (Brenman et al., 1996; Hölscher, 1997; Contestabile, 2000; Calabrese et al., 2007; Garthwaite, 2008; Steinert et al., 2010), the loss of NOS1 in the developing FXS neocortex may contribute to the pathophysiology of the disorder. Here, we assess disrupted NO signaling as a candidate mechanism underlying FXS, focusing on the highly specific spatiotemporal expression pattern of NOS1 in the fetal human neocortex and the potential neurobiological contribution of disrupted NO signaling to FXS and other neurodevelopmental disorders.

## SPATIOTEMPORAL AND SPECIES-DEPENDENT EXPRESSION OF NOS1 IN THE HUMAN NEOCORTEX

**FIGURE 3:**
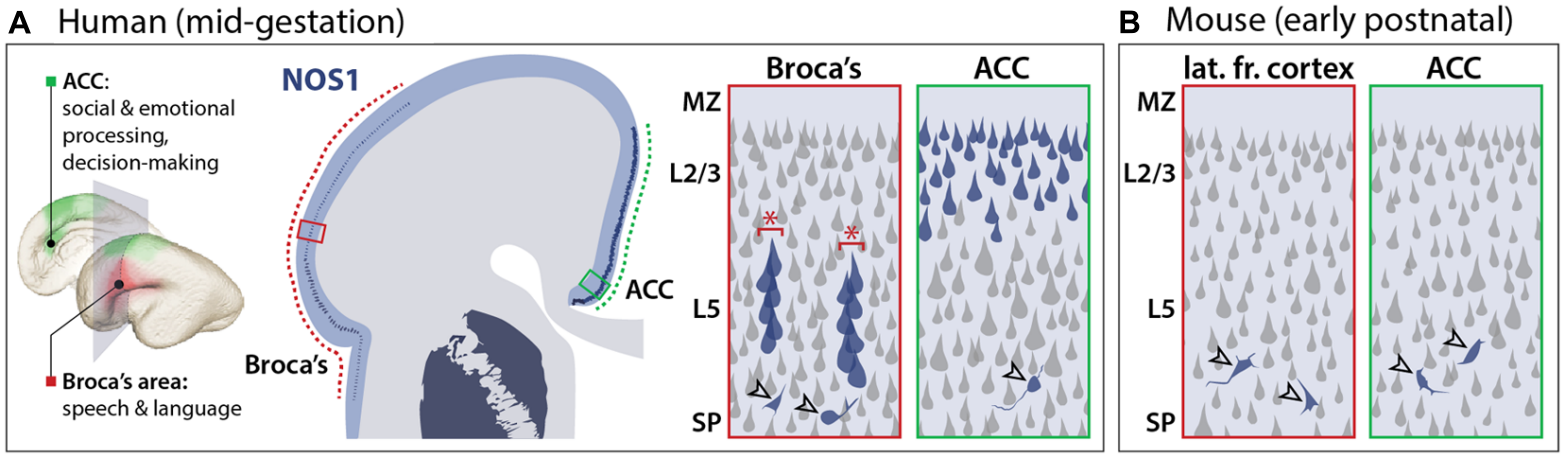
**Species-dependent NOS1 expression in the developing neocortex. (A)** In the mid-fetal human neocortex, NOS1 is specifically expressed in the pyramidal neurons of two neocortical regions underlying language and cognitive functions. In the Broca’s area (red boxes), NOS1 expression (blue), is selectively present in the pyramidal neurons of neocortical layer 5 (L5). NOS1-expressing neurons are assembled into minicolumns (red asterisks) separated by NOS1-negative neurons. In the anterior cingulate cortex (ACC; green boxes), many L2/L3 pyramidal neurons express high levels of NOS1. NOS1 expression in interneurons (open arrowheads) is present throughout neocortical areas and layers with no significant selectivity. **(B)** In the early-postnatal mouse neocortex, which is developmentally equivalent to the human mid-fetal neocortex, NOS1 is exclusively expressed in interneurons (open arrowheads) and absent from pyramidal neurons.

To determine the potential contribution of NOS1 to FXS, it is necessary to consider the time and location of NOS1 function during brain development. In contrast to the widespread expression of FMRP, NOS1 expression exhibits spatial, temporal, and neuronal subtype specificity, providing insights into its potential neural function. In the neocortex of several mammalian species, NOS1 has been known to be a selective marker of inhibitory interneurons (Vruwink et al., 2001) while being absent from the principal neurons of the neocortex, the excitatory projection (pyramidal) neurons. In the human mid-fetal (13–24 weeks post-conception; **Figure 1**) neocortex, however, we found NOS1 to be present not only in the interneurons, but also in the pyramidal neurons of two specific neocortical areas underlying higher cognitive functions: the fetal Broca’s area and orofacial motor cortex, and the ACC (**Figure 3**; Kwan et al., 2012a). The pyramidal projection neurons occupy a central position in neocortical circuitry, being the source of long distance axonal projections that connect distal areas within the neocortex (via intracortical connections) and the neocortex with subcortical brain structures (via corticofugal connections). The newly identified pyramidal expression of NOS1 is particularly surprising because pyramidal neurons are derived from distinct progenitors and have greatly divergent chemical and electrophysiological properties compared to interneurons (Markram et al., 2004; Kwan et al., 2012b). Interestingly, the expression of NOS1 in the human neocortex sharply contrasts that in the mouse neocortex, wherein NOS1 is exclusively present in inhibitory interneurons (**Figure 3**; Kwan et al., 2012a). NOS1 expression analysis in the fetal macaque neocortex and sequence conservation analysis in mammals revealed that pyramidal NOS1 expression is likely to be primate-specific. The recent origin of this expression pattern is consistent with the possibility that pyramidal NOS1 plays a key role in the development or function of neocortical circuitry underlying higher cognitive functions.

In addition to this notable spatial selectivity, NOS1 expression in the human neocortex further exhibits remarkable temporal specificity, occurring only transiently within a small mid-fetal window and becoming dramatically downregulated by late gestation (Figures 1 and 3). During this mid-gestation period, the genesis of neocortical pyramidal neurons is largely complete and the deep neocortical layers have been formed, whereas many upper layers neurons are migrating toward their laminar destination. Importantly, neocortical axon tracts are undergoing outgrowth to reach their target destinations, a process that largely precedes the onset of peak synaptogenesis (Hadley et al., 1983; Kang et al., 2011; Xu and Henkemeyer, 2012). Overall, the spatiotemporal specificity of pyramidal NOS1 expression is consistent with a key role for NOS1 in the formation of specific neocortical circuits important for higher cognition. The location, connectivity, and neural function of these circuits provide crucial insights into the potential consequences of NOS1 dysregulation in the FXS neocortex.

## BROCA’S SPEECH AND LANGUAGE AREA AND OROFACIAL MOTOR CORTEX

In the mid-gestation frontal and frontoparietal operculum, the fetal anlage of the adult Broca’s speech and language area and orofacial motor cortex, NOS1 is specifically expressed in layer 5 (L5) corticofugal projection neurons, the output neurons of the neocortex (**Figure 3**; Kwan et al., 2012a). Broca’s area, located in the inferior frontal gyrus, plays a key role in the generation of speech and is also important in language comprehension (Judas and Cepanec, 2007; Keller et al., 2009). The L5 expression of NOS1 in the fetal Broca’s area is developmentally regulated, being exclusively present from approximately 16 to 24 weeks post-conception. The expression of NOS1 in these neurons during the 7–8 week fetal window when they differentiate and acquire their axonal connectivities implies a role for NOS1 in the assembly of motor circuits involved in speech and language. Several lines of evidence, including studies of gene co-expression, microscopic neuroanatomy, and functional imaging, point to a putative contribution of disrupted NOS1 expression in Broca’s area to the psychopathology of FXS and provide insights into the potential mechanistic underpinnings of this disruption.

In the mid-fetal Broca’s area, NOS1 neurons coexpress BCL11B (previously CTIP2), a transcription factor that is specifically expressed in and required for the development of corticofugal projection neurons (Arlotta et al., 2005). This coexpression is consistent with the L5 pyramidal identity of NOS1 neurons and a potential role for NOS1 in neocortical output circuits originating from this area. Abnormal activation of the inferior frontal gyrus, which encompasses the Broca’s area, in FXS patients during the counting Stroop interference task (Tamm et al., 2002) has suggested altered Broca’s area connectivity in the disorder. Furthermore, functional imaging studies of ASD patients have identified specific alterations in Broca’s area connections. For example, in a recent analysis of network connectivity in 24-month old ASD infants, alterations in network efficiencies were reported in all four cortical lobes, with a significant reduction in the Broca’s area in the frontal lobe (Lewis et al., 2014). Importantly, reduced network efficiency in Broca’s area correlated with increased ASD symptom severity. In another recent study, naturally sleeping ASD toddlers exhibited significantly weakened interhemispheric correlations in the inferior frontal gyrus (Dinstein et al., 2011). Interestingly, the strength of the interhemispheric synchrony was negatively correlated with autism severity and positively correlated with verbal ability. Furthermore, a reversal in volume asymmetry in the Broca’s area has been reported in autistic boys (De Fossé et al., 2004), implicating both structural and functional differences in this area in ASD. It remains to be shown whether NOS1 contributes to Broca’s area alterations in ASD or similar alterations in FXS. The highly specific spatiotemporal expression of NOS1, however, is consistent with this possibility.

In the mid-fetal Broca’s area, NOS1 neurons coexpress FOXP2 (Kwan et al., 2012a), a gene altered in a severe speech disorder (Lai et al., 2001; Fisher and Scharff, 2009) and implicated in ASD and cognitive impairments (Bacon and Rappold, 2012). The presence of NOS1 in FOXP2-expressing neurons adds further support to a possible role of NOS1 in ASD and cognitive neural circuits. Furthermore, NOS1-expressing neurons are organized into microcircuits known as minicolumns (**Figure 3**; Kwan et al., 2012a), a vertical arrangement thought to constitute a functional unit of cortical circuitry (Mountcastle, 1997; Buxhoeveden and Casanova, 2002). Interestingly, the arrangement of minicolumns is disorganized in psychiatric disorders, including ASD and SCZ, potentially contributing to circuit dysfunction by disrupting the inhibitory architecture of the neocortex (Buxhoeveden and Casanova, 2002; Casanova et al., 2002, 2003). The expression of NOS1 in minicolumns during fetal development is consistent with a role for NO in the assembly of microcircuitry, possibly related to ASD and SCZ, in this distinctly human neocortical area.

## ANTERIOR CINGULATE CORTEX

In addition to the Broca’s area, a second neocortical region with important cognitive functions also exhibit pyramidal NOS1 expression during mid-fetal development. Starting from approximately 16 weeks post-conception, NOS1 is expressed in a large number of upper-layer (L2–L3) intracortical projection neurons of the ACC at significantly higher levels compared to the L5 neurons of the Broca’s area (Kwan et al., 2012a). NOS1-expressing neurons in the ACC, in contrast to those in the Broca’s areas, are not marked by BCL11B but instead coexpress SATB2, a transcription factor that labels intracortical projection neurons and is required for their normal development (Alcamo et al., 2008; Britanova et al., 2008). Unlike the corticofugal projection neurons that express NOS1 in the Broca’s area, NOS1-expressing pyramidal neurons in the ACC are likely to contribute to intracortical connectivity. Furthermore, although NOS1 expression in ACC pyramidal neurons is also temporally regulated, its timing spans a lengthier period during development, being present at high levels from mid- to late- gestation and downregulated after birth (Kwan et al., 2012a). Whereas no pyramidal neurons retain NOS1 expression in the postnatal Broca’s area, a small number of pyramidal neurons in the ACC express low levels of NOS1 into adulthood (Gao et al., 2013). The significance of NOS1 expression in adult pyramidal neurons and whether its function parallels its fetal role remains to be explored. The ACC is not only highly interconnected to cognitive and emotional circuits, but also important in a number of neuropsychiatric diseases (Devinsky et al., 1995). Functional imaging, gene expression analyses, and other studies suggest the possibility that NOS1-expressing pyramidal neurons in the ACC may contribute to the psychopathology of FXS in a manner distinct from their counterparts in the Broca’s area.

Centrally positioned in the neocortex, the ACC is a key point of integration for cognitive and emotional processes, playing a crucial role in executive, social, and affective functions (Devinsky et al., 1995). The ACC exhibits extensive intracortical connectivities. The dorsal cognitive portion of the ACC is connected with motor and sensory systems of the neocortex, and is therefore well-positioned to integrate sensory stimuli with top-down commands (Paus et al., 1993; Picard and Strick, 1996; Williams et al., 2004; Crottaz-Herbette and Menon, 2006). The ventral part of the ACC, which is involved in emotion and motivation processing, is connected with the anterior insula and amygdala, as well as the nucleus accumbens and hypothalamus (Bush et al., 2002; Etkin et al., 2011). Functional imaging studies have found the ACC to be important for performance monitoring, error detection, action selection, and reinforcement of adaptive behavior (Brown and Braver, 2005; Schweimer and Hauber, 2006; Ko et al., 2009). Alterations in ACC circuitry have been proposed to contribute to maladaptive behaviors in psychiatric disorders (Drevets et al., 2008; Davey et al., 2012; Tu et al., 2012; Zikopoulos and Barbas, 2013). The expression of NOS1 in the intracortical projection neurons of the ACC implicates NOS1 in the connectivity of cognition and emotion circuits, which likely underlie some aspects of the pathogenesis in a number of neuropsychiatric disorders.

Data from several studies lend indirect support to a potential role of ACC NOS1 dysregulation in FXS. Studies of a human *NOS1* hypomorphic allele revealed homozygosity to be associated with attention deficit hyperactivity disorder (ADHD), impulsivity, and aggression (Reif et al., 2009), linking reduced NOS1 activity to behavioral features often comorbid with FXS (Rogers et al., 2001). This *NOS1* hypomorphism has also been linked to reduced neural activity in the ACC (Reif et al., 2009), consistent with a potential role of NOS1 in the development of ACC connectivity. Additional functional imaging studies revealed a similar reduction in ACC activation in FXS and ADHD patients during attentional processing tasks (Bush et al., 1999; Uddin and Menon, 2009) and in autistic children in response to a familiar face (Pierce and Redcay, 2008). The convergence of neural and behavioral deficits between *NOS1* hypomorphism, FXS, and FXS comorbidities provide robust support to the possibility of a functional role of NOS1 in the development and function of ACC circuitry related to FXS and ASD.

In addition to FXS and ASD, ACC dysfunction has also been associated with other psychiatric disorders. In SCZ, pathological studies have found neuronal architecture to be altered in the ACC (Salgado-Pineda et al., 2014), whereas neural imaging studies have found abnormalities in the ACC that were reversed with treatment response. In major depressive disorder, neuronal density was found to be reduced in the ACC (Rajkowska et al., 1999, 2001) and psychotherapy success was found to correlate with increased ACC activity (Mayberg et al., 1997; Quidé et al., 2012). Interestingly, a recent study of depressive patient materials revealed a significant reduction in NO metabolites in the cerebrospinal fluid (Gao et al., 2013). Furthermore, NOS1 expression in the pyramidal neurons of the adult ACC was found to be significantly reduced in depressed patient brains (Gao et al., 2013). Although FXS has not been associated with major depression, *de novo* variations in SCZ have been shown to preferentially affect FMRP targets and FMRP levels have been correlated with SCZ severity (Kovács et al., 2013; Fromer et al., 2014; Purcell et al., 2014), suggesting a potential mechanistic overlap.

The spatiotemporal expression pattern of NOS1 during circuit wiring is consistent with a potential role in establishing global connectivity during neocortical development. By imaging resting state coordinated activity between brain areas, a measure of functional connectivity can be inferred based on synchronous activity (Power et al., 2011). Remarkably, Broca’s area and the ACC, the two neocortical areas with significant mid-fetal pyramidal NOS1 expression, exhibit coordinated activity and are known to form a cingulo-opercular network (Power et al., 2011), one of the default mode networks of the neocortex (Vaden et al., 2013; Sestieri et al., 2014). Interestingly, a disruption in the activity of the cingulo-opercular network has been identified in SCZ (Tu et al., 2012). Although the cingulo-opercular network has not been examined in individuals with the hypomorphic *NOS1* allele, these data suggest a potential contribution of reduced NOS1 activity to the altered connectivity of a default mode network. Together, these findings converge on the possibility that NOS1 dysfunction in pyramidal neurons of the ACC and Broca’s area may contribute to distinct psychiatric disorders that affect higher cognitive abilities.

## A FUNCTIONAL CONVERGENCE ON SYNAPTIC MECHANISMS

Recent advances in psychiatric genetics have reaffirmed the heterogeneity of disease loci and the complexity of these disorders. However, a functional convergence has emerged from many of these studies. In ASD, specific functional subclasses of risk loci have been identified based on gene ontology and network analysis (Krumm et al., 2014; Pinto et al., 2014). These unbiased analyses have revealed chromatin biology and synaptic function to be key processes in ASD. Furthermore, empirical evidence has also indicated that the behavioral and intellectual deficits present in ASD, and often comorbid with FXS, likely reflect an underlying defect in synaptic development, plasticity, or function (Zoghbi, 2003; Garber, 2007). Interestingly, similar analyses of SCZ genes have identified a number of shared risk loci, particularly in chromatin modifiers and synaptic molecules (McCarthy et al., 2014). The emerging data have shown many of the synaptic genes associated with ASD or SCZ to encode mRNA transcripts regulated by FMRP (Darnell et al., 2011; Iossifov et al., 2012; Fromer et al., 2014; Purcell et al., 2014). Furthermore, synaptic dysregulation is now recognized as a key pathology in a number of psychiatric disorders, suggesting the synapse to be a common neural substrate underlying distinct clinical manifestations of cognitive impairment (Penzes et al., 2011).

From the earliest functional studies, it has been clear that FXS involves deficits at the synapse. Alterations in spine morphology is well-characterized in human FXS (Comery et al., 1997) and animal models of FXS (Irwin et al., 2000; Nimchinsky et al., 2001), with both presenting significantly more morphologically immature spines and fewer mature spines. Similar deficits in spine morphology and synapse function have been reported to be present in ASD (Kaufmann and Moser, 2000; Hutsler and Zhang, 2010; Penzes et al., 2011). The localization of FMRP to dendritic spines and the putative function of many of its target mRNAs at or near synaptic contacts add further support to the possibility that FXS is a disorder of synaptic development and function (Irwin et al., 2000; Huber et al., 2002; Bear et al., 2004).

**FIGURE 4:**
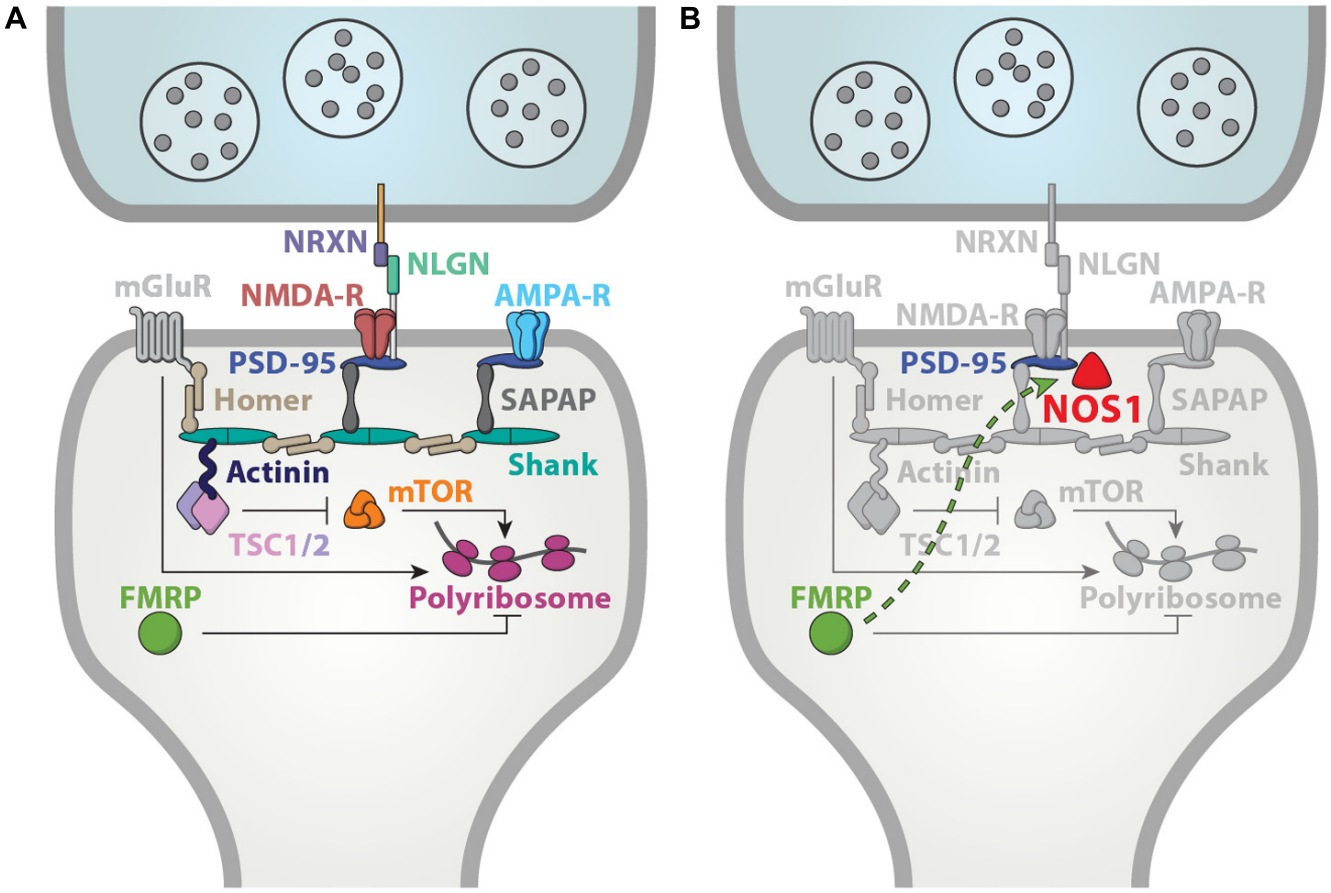
**A functional convergence on synaptic mechanisms. (A)** Many ASD-associated genes are localized to the synapse, robustly implicating synaptic dysfunction in ASD. Adapted from Peça et al. (2011). **(B)** NOS1 is anchored to the post-synaptic member via interactions with PSD-95 (Brenman et al., 1996), and therefore, well-positioned to contribute to the synaptic mechanisms underlying FXS and ASD.

Consistent with a putative contribution of dysregulated NO signaling to FXS pathophysiology, NOS1 is likely to have a synaptic function as well. Subcellularly, NOS1 is significantly localized to the dendritic spines (Burette et al., 2002; Kwan et al., 2012a). This localization is mediated by direct interaction with the PDZ domain of post-synaptic density protein PSD-95 (Brenman et al., 1996). The catalytic activity of NOS1 requires Ca^2+^ influx, which is under the control of near by NMDA receptors (Brenman and Bredt, 1997; Sattler et al., 1999)_._ Interestingly, blockade of NOS1 function has been shown to disrupt synapse formation and result in spine loss (Sánchez-Islas and León-Olea, 2004; Morales-Medina et al., 2007; Nikonenko et al., 2008). Therefore, aspects of spine morphology deficits in FXS may originate from a loss of NO signaling. Overall, given the key role of synaptic proteins in ASD, NOS1 is well positioned to mediate at least some of the mechanisms disrupted in FXS (**Figure 4**).

## NITRIC OXIDE SIGNALING AS A TARGET FOR FXS MOLECULAR THERAPIES

The development of molecular therapeutic interventions for neurodevelopmental disorders represents an immense challenge. In the past decades, these efforts have been impeded by the tremendous phenotypic and genotypic heterogeneity in the patient populations, the lack of specific knowledge on the molecular pathways, brain regions, or developmental timepoints most relevant to the pathophysiology of the disorders, and the identification of the appropriate primary endpoints (Haddad and Dursun, 2008; Jacquemont et al., 2014). Recent advances in psychiatric genetics have helped to pinpoint some of the molecular and neural underpinnings of these disorders, and revealed potential targets for therapeutic interventions. These strategies have focused mainly on alterations in molecular signaling pathways, which may be more targetable compared to structural deficits in brain development.

Since the proposal of the mGluR theory (Bear et al., 2004; Pop et al., 2014), a number of therapies targeting molecules up or downstream of mGluR signaling have been developed and reached various phases of clinical trial. For example, an open label trial of fenobam (NPL-2009), an mGluR5 antagonist, ameliorated anxiety, hyperarousal, and deficits in prepulse inhibition, and improved continuous performance task outcomes (Berry-Kravis et al., 2009). Arbaclofen (STX209), a GABA receptor agonist acting upstream of mGluR signaling, showed mixed promise in correcting behavioral problems, which was strengthened when adjusted for baseline severity of social withdrawal (Berry-Kravis et al., 2012; Erickson et al., 2014; Jacquemont et al., 2014). The mGluR antagonist mavoglurant (AFQ056) has shown some promise in patients with complete methylation at the *FMR1* promoter and in an animal model of FXS (Jacquemont et al., 2011; Gantois et al., 2013). The commercial futures of arbaclofen and mavoglurant, however, are unclear, due to the recent discontinuation of their development.

The possibility of dysregulated NO signaling in FXS may lead to novel therapeutic strategies, including those that increase the brain levels of NO or cyclic guanosine monophosphate (cGMP), a second messenger elevated downstream of NO-mediated activation of soluble guanylate cyclase (sGC; Garthwaite and Boulton, 1995). Phosphodiesterase (PDE) inhibitors and NO donors have been used to enhance cGMP at distinct points of the NO signaling pathway. PDE type 5 inhibitors, which elevate cGMP levels by blocking their enzymatic degradation, are well known for their role in treating erectile dysfunction (Boolell et al., 1996; Sandner et al., 2008), but may have additional indications in the brain. Sildenafil has been shown to have antidepressant, anxiolytic, and cognitive effects, and to increase cGMP levels in the hippocampus (Rutten et al., 2005; Liebenberg et al., 2010, 2012; Uthayathas et al., 2013; Zhang et al., 2013). Tadalafil has similar antidepressant and anxiolytic effects, and has additionally been shown to reverse cognitive dysfunction by crossing the blood–brain barrier (Liebenberg et al., 2010, 2012; García-Barroso et al., 2013). cGMP levels may also be increased using NO donors to stimulate sGC activity. One such donor is sodium nitroprusside, a single intravenous administration of which has been shown to rapidly ameliorate the positive, negative, anxiety, and depressive symptoms in SCZ patients (Hallak et al., 2013). Interestingly, NO donors may mediate effects in addition to sGC activation. For example, the NO donor minocycline is known to inhibit matrix metalloproteinase-9 (MMP9), a protein encoded by an FMRP target mRNA (Berry-Kravis, 2014). It also promotes spine maturation and normal behavior in *Fmr1* null mice (Bilousova et al., 2009) and delivers a significant improvement in anxiety and mood-related behaviors in children diagnosed with FXS (Paribello et al., 2010; Leigh et al., 2013). Additional drugs may more directly target NOS1 function or expression. For example, sapropterin, a synthetic form of the NOS cofactor tetrahydrobiopterin, was found to improve behavioral symptoms of ASD (Frye et al., 2013). A drug currently undergoing clinical trials for FXS and Rett syndrome may also affect NOS expression. NNZ-2566, a GPE peptide analog has been previously demonstrated to increase neuronal ChAT, GAD, and NOS expression (Guan et al., 1999; Guan and Gluckman, 2009; Berry-Kravis, 2014). Further studies on these and other therapies targeting NOS are expected to reveal the feasibility and efficacy of this line of treatment strategy for FXS and ASD.

## NO SIGNALING IN MULTIPLE PSYCHIATRIC DISORDERS

Emerging genetic studies of multiple psychiatric disorders previously thought to be mechanistically distinct have implicated the same set of genes to carry disease risk for each disorder. A recent analysis of *de novo* variants uncovered shared genetic underpinnings in ASD and SCZ (McCarthy et al., 2014). In a recent large genome-wide association study (GWAS), risk is correlated across five psychiatric disorders: ASD, SCZ, attention deficit-hyperactivity disorder, bipolar disorder, and major depressive disorder (Smoller et al., 2013). Remarkably, from this pooled analysis, single nucleotide polymorphisms (SNPs) within two L-type voltage-gated calcium channel (VGCC) subunits, *CACNA1C* and *CACNB2*, surpassed statistical threshold. This and previous studies associating *CACNA1C* with the individual disorders [Splawski et al., 2006; Ferreira et al., 2008; Walsh et al., 2008; Schizophrenia Psychiatric Genome-Wide Association Study (GWAS) Consortium, 2011; Lu et al., 2012; Wray et al., 2012] strongly implicate VGCC signaling to be a shared neurobiological mechanism between these phenotypically divergent illnesses.

The genetic and mechanistic evidence presented in this review suggest that NOS1 may similarly play a role in multiple psychiatric disorders. NOS1 activity is dependent on calcium signaling mediated via calmodulin. This calcium signaling may partially originate from L-type VGCCs. One study found depolarization-stimulated and spontaneous cGMP formation downstream of NOS1 activity to be blocked by the L-type calcium channel blocker nifedipine (Oka et al., 1999). Furthermore, there is evidence that NOS1, in turn, regulates VGCCs including CACNA1C. The protein levels of CACNA1C and CACNA1D were found to be significantly reduced in the cerebellum of *Nos1* null mice (Kim et al., 2004). Together, these data suggest that the potential involvement of NOS1 in multiple psychiatric disorders may be mediated in part via interactions with VGCCs.

The pursuit of convergent mechanisms in multiple psychiatric disorders may help identify points at which disease pathways intersect and may lead to a more mechanistic nosology in psychiatry beyond descriptive classification. These studies are expected to not only lead to conceptual advances in the neurobiology of psychiatric disorders, but also to targets of molecular therapies with widespread utility. In particular, because NO signaling has been targeted pharmacologically for other indications, understanding NOS1 dysfunction in psychiatric disorders has tremendous potential to lead to the repurposing of efficacious, tested agents to treat these disorders.

## Conflict of Interest Statement

The authors declare that the research was conducted in the absence of any commercial or financial relationships that could be construed as a potential conflict of interest.
